# Influence of Elicitation and Drying Methods on Anti-Metabolic Syndrome, and Antimicrobial Properties of Extracts and Hydrolysates Obtained from Elicited Lovage (*Levisticum officinale* Koch)

**DOI:** 10.3390/nu13124365

**Published:** 2021-12-04

**Authors:** Anna Jakubczyk, Urszula Złotek, Kamila Rybczyńska-Tkaczyk

**Affiliations:** 1 Department of Biochemistry and Food Chemistry, University of Life Sciences in Lublin, Skromna 8, 20-704 Lublin, Poland; anna.jakubczyk@up.lublin.pl (A.J.); urszula.zlotek@up.lublin.pl (U.Z.); 2 Department of Environmental Microbiology, University of Life Sciences in Lublin, Leszczyńskiego Street 7, 20-069 Lublin, Poland

**Keywords:** lovage, elicitors, drying method, anti-metabolic syndrome, antimicrobial properties

## Abstract

This research aims to investigate the influence of elicitation and drying methods (natural, convection, microwave, and freeze-drying), with jasmonic acid (JA) and yeast extract (YE) on the biological activity of extracts and hydrolysates from lovage (*Levisticum officinale* Koch) leaves. The results indicate that the highest TPC was determined for hydrolysates obtained from JA-elicited microwave-dried lovage (24.96 mg/gDW). The highest ACE and lipase inhibitory activity was noted for PBS extract obtained from JA-elicited lovage after microwave drying (EC_50_ = 0.16 and 0.12 mg/mL, respectively). Ethanolic extract from JA-elicited lovage after freeze-drying was characterized by the highest α-amylase inhibitory activity (EC_50_ = 3.92 mg/mL) and the highest α-glucosidase inhibitory activity (EC_50_ = 1.43 mg/mL) was noted for hydrolysates from control plants subjected to freeze-drying. The highest antimicrobial activity towards *C. albicans* yeasts was observed for microwave ethanolic extracts with minimal inhibition (MIC) and lethal (MLC) concentrations of 0.625 and 1.25 mg/mL, respectively.

## 1. Introduction

Nowadays, consumers pay attention to food that is rich in health-improving nutrients and bioactive ingredients. Natural compounds from plants have been used in functional foods as an alternative to synthetic additives, which are now not acceptable to consumers. Herbs that have been used in medicine and therapy for a long time are now one of the most common groups of food ingredients. Moreover, they are responsible for the taste and aroma of dishes and may improve the composition of food and often the bioavailability, in addition to the bioactivity of essential and active ingredients [1].

Lovage (*Levisticum offcinale* L.) is an aromatic perennial herbaceous plant from the *Apiaceae* family known for centuries as an agent used in the treatment of many diseases due to its diuretic, carminative, and spasmolytic activity. It originates from West Asia but also grows in North America and many European countries [2]. The bioactive compounds obtained from the seeds, leaves, and roots of this plant have been used in many industries, especially in food, perfumery, tobacco, or beverage manufacture. It should be emphasized that it is most commonly used in the food industry, particularly for condiment production, due to its characteristic taste and aroma, giving many dishes their original flavor [3]. Lovage is a source of many bioactive compounds, mainly essential oils, coumarins, alkaloids, polyacetylenes, and polyphenols (phenolic acids, flavonoids, lignans, stilbenes, curcuminoids, tannins, quinones, and many others). These compounds determine the bioactive properties of the plant, and their functions are mainly associated with antioxidant and anti-inflammatory activity, free radical scavenging, and the regulation of many enzymes [4].

One of the most common diseases associated with lifestyle disorders is metabolic syndrome (MS). It is a complex of components that are associated with overnutrition and may be described as central obesity, high blood pressure, disorders in glucose content in blood, high blood glucose, inflammatory processes, hypertriglyceridemia, and hypercholesterolemia. Generally, MS increases the risks of type 2 diabetes, cardiovascular disease, stroke, and mortality [5]. In addition to pharmacological treatment, patients with MS should primarily change their lifestyle by increasing their physical activity and changing their eating habits. Their diet should be rich in compounds that inhibit the activity of enzymes involved in MS development, such as pancreatic lipase, angiotensin-converting enzyme (ACE), α-amylase, or α-glucosidase. Phenolic compounds of plant origin have been well described as inhibitors of these enzymes and may be used to support pharmacological therapy for MS. Moreover, many studies have indicated that the antimicrobial activity of the plant material influences immunity and may inhibit inflammatory processes.

The content of bioactive compounds in plant material is influenced not only by the part of the plant from which the compounds are obtained but also by the method of preserving and storing the plants. One of the main methods of preservation of plants is drying. There are many drying methods used in agricultural crops and the food industry, e.g., microwave, heat-stream, vacuum, freeze, hot-air, and convection drying. The choice of the drying method depends on the type of product and its bioactive ingredients. The most common drying methods are open-air drying or solar drying. These methods are cheap but need a lot of time [6], and this is the reason for searching for new optimal drying methods to preserve food plants.

The main aim of this study was to investigate the influence of elicitation and drying methods on the biological activity of extracts and hydrolysates from lovage leaves. Elicitation is one of the methods used to improve plant resistance and induce the production of secondary metabolites. This natural method for the induction of plant resistance mechanisms and the synthesis of phytochemical compounds is especially important in food production. In this study, jasmonic acid and yeast extract were used as elicitors.

## 2. Materials and Methods

### 2.1. Research Material

The research material used was leaves of lovage (*Levisticum officinale* Koch. cv. Elsbetha) obtained from the Enza Zaden Company (Enkhuizen, The Netherlands), grownin controlled elicitation conditions.

#### 2.1.1. Plant Growth Conditions

The lovage was grown in a chamber (SANYO MLR-350H) in conditions described by Złotek et al. [7]. Twenty-day-old plants were elicited with 10 µM jasmonic acid (JA) and 0.1% yeast extract (YE) water solutions. Tween 20 was used as a surfactant. The most effective concentrations of elicitors were selected based on a previous study [7]. The control (C) comprised lovage plants without elicitation. Twenty-five days after the elicitation, the plants were collected and dried via four methods: natural drying, convection drying, microwave drying, and freeze-drying. The dried plants were used in further analysis. The experiments were conducted in triplicate.

#### 2.1.2. Drying Method

Fresh herbs were dried using four techniques: natural drying (in a dark room at a temperature between 20 °C to 22 °C for approximately 7 days), convection drying (in a drying oven at 40 °C for approximately 5 h), microwave drying (in a laboratory microwave dryer, microwave power 360 W at 20 °C for ca. 5 min), and freeze-drying (in a lyophilizer at a temperature of −49 °C and pressure of 0.045 mbar).

### 2.2. Preparation of Samples

#### 2.2.1. Ethanolic Extracts

Ethanolic extracts were prepared for further analysis (0.3 g DWin 15 mL of 70% (*v*/*v*) acidified ethanol (0.1% HCl), sonication at 30 °C for 1 h, and centrifugation at 9000× *g* for 30 min).

#### 2.2.2. PBS Extracts

For the preparation of buffer extracts, dried leaf tissue (0.5 g) was homogenized, extracted for 30 min with 20 mL of PBS buffer (phosphate buffered saline, pH 7.4), and centrifuged at 9000× *g* for 20 min. Next, the residues were extracted again with 20 mL of the PBS buffer. The supernatants were combined and adjusted to a final volume of 50 mL with PBS buffer.

#### 2.2.3. Hydrolysates

In vitro digestion was performed as described previously by Gawlik-Dziki et al. [8].

### 2.3. Total Phenolic Content (TPC)

The number of total phenolics was determined in ethanolic, PBS extracts, and hydrolysates using a Folin–Ciocalteau reagent [9]. The total phenolic content was calculated as a gallic acid equivalent (GAE) μg per g of dry weight (DW).

### 2.4. Inhibitory Effect of Samples on Enzymes Involved in Metabolic Syndrome

#### 2.4.1. ACE Inhibition

The angiotensin-converting enzyme was prepared according to the method described by Karaś et al. [10].

#### 2.4.2. Pancreatic Lipase Inhibitory

Lipase inhibitory activity was determined with the method described by Jakubczyk et al. [11]. The final volume of the reaction mixture was 150 μL (2 μL of the enzyme, 5 μL of the sample, 142 μL of 100 mM potassium phosphate buffer, pH 7.5, and 1 μL of a 100 mM *p*-nitrophenyl acetate (pNPA) solution in dimethyl sulfoxide (DMSO)). Changes in absorbance at 234 nm were measured after 5 min using BioTek Microplate Readers (Epoch™ 2 BioTek, Bad Friedrichshall, Germany).

#### 2.4.3. Potential Anti-Diabetic Effect

##### α-Amylase Inhibitory Activity

α-Amylase inhibitory activity (αAI) of the samples was measured according to the method described by Świeca, Baraniak, and Gawlik-Dziki [12].

##### α-Glucosidase Inhibitory Activity

α-Glucosidase inhibitory activity was determined with the use of a sucrose solution as a substrate according to the following method described by Karaś et al. [10].

### 2.5. Antimicrobial Effect

The samples were tested against bacteria *Escherichia coli* ATCC 25922, *Staphylococcus aureus* ATCC 29737, *Listeria monocytogenes* ATCC BBA-2660, *Bacillus cereus* ATCC 14579, *Salmonella enteritidis* ATCC 4931, and the yeast *Candida albicans* ATCC 90028. These strains were obtained from the American-Type Culture Collection (ATCC, distributors: LGC Standards, Łomianki, Poland) and stored at 4 °C. All strains were cultured at 37 °C on a nutrient broth (NB) medium.

#### 2.5.1. Determination of Minimum Inhibitory Concentration (MIC) and Minimum Lethal Concentrations (MLC)

Serial twofold dilutions of each sample were made with Mueller Hinton Broth (MHB) to yield final concentrations ranging from 5 to 0.312 mg mL^−1^ and placed in 96-well plates. Then, 100 μL of bacterial (10^8^ CFU mL^−1^) or yeast (10^5^ CFU mL^−1^) culture was added. Wells with MHB or bacterial culture were negative and positive controls, respectively. Penicillin G and streptomycin (250–0.24 µg mL^−1^), as well as cyclohexamide (500–0.48 µg mL^−1^), were used as positive controls for the bacterial and yeast cultures, respectively. Minimum lethal (MLC) concentrations for bacteria and yeast were determined after broth microdilution by subculturing a sample from the wells showing no microbial growth on the surface of the Mueller Hinton Agar (MHA) medium. The plates were incubated at 37 °C for 18 h. The MLC was defined as the lowest concentration of the antimicrobial agent needed to kill 99.9% of the final inoculum after 18h of incubation.

#### 2.5.2. Biotoxicity Assay Using Resazurin Reduction Method

Resazurin reduction assays were performed according to Jakubczyk et al. [4] to estimate biotoxicity against bacteria and yeast.

### 2.6. Statistical Analysis

All determinations were performed in triplicate. Statistical analysis was performed using STATISTICA 13.1 software for a mean comparison using ANOVA, with post-hoc Tukey’s honestly significant difference (HSD) test at a significance level of α = 0.05.

## 3. Results

### 3.1. Phenolic Content in Samples

Phenolic compounds are one of the most important groups in lovage leaves. In our study, we determined the influence of the drying method and the elicitation effect on phenolic compounds in two types of extract: ethanolic and PBS, in addition to hydrolysates obtained after in vitro digestion in gastrointestinal conditions. As shown in Table 1, both tested factors had an influence on phenolic compounds. The highest phenolic content in the ethanolic extract was noted in samples elicited with jasmonic acid after freeze-drying (18.48 mg/gDW). In turn, the highest content of phenolic compounds in the PBS extract was determined for samples after elicitation with jasmonic acid and drying with the convection method (14.52 mg/gDW). It should be noted that this content did not show statistically significant differences with the other values. The highest concentration of phenolic compounds was determined in the hydrolyzates. The highest value was characteristic for samples obtained after elicitation of the plants with jasmonic acid and microwave- and freeze-drying (24.96 and 23.76 mg/gDW, respectively). The data indicated that the elicitation of the plants with 10 µM jasmonic acid had the greatest influence on the phenolic content determined in different samples of lovage leaves.

### 3.2. ACE and Lipase Inhibitory Activity of Samples

Metabolic syndrome is characterized by high blood pressure and obesity. These two disorders are caused by poor eating habits, which lead to excessive activity of enzymes such as ACE and lipase. The results of this experiment indicated that both drying methods and elicitation had an influence on the inhibition of these enzymes (Table 2). This activity was expressed as EC_50_ (mg/mL) values. The highest ACE inhibitory activity was determined in the PBS extract obtained from jasmonic acid-elicited plants subjected to microwave drying (EC_50_ = 0.16 mg/mL). It should be noted that this activity was not detected in all samples. As indicated by the presented data, most of the PBS extracts and hydrolysates of plants dried with the natural and convection method did not exhibit ACE-inhibitory activity. Moreover, no PBS extracts from the freeze-dried plant material had ACE inhibitory activity. In the group of the hydrolysates, only the control sample did not exhibit such activity. In this case, the elicitation had an influence on the inhibition activity of the samples, and the highest ACE inhibitory effect was noted in hydrolysates obtained from plants elicited with jasmonic acid (EC_50_ = 1.32 mg/mL).

The elicitation and drying method also had an influence on the lipase inhibitory activity of tested samples. The highest lipase inhibitory effect was determined in the PBS extract obtained from plants elicited with jasmonic acid and subjected to microwave drying (EC_50_ = 0.12 mg/mL). It should be noted that the drying method had the greatest influence among the ethanolic extracts, as this activity was not determined in all samples. No lipase inhibitory activity was detected in all samples dried with the natural method. This activity was detected only in extracts from plants elicited with yeast extract and subjected to convection drying (lipase inhibitory EC_50_ value of 0.15 mg/mL). However, in the group of samples treated with the microwave drying method, the highest lipase activity was determined in the control sample (EC_50_ = 0.17 mg/mL), but it was not detected in the ethanolic extract obtained from yeast extract-elicited plants.

### 3.3. α-Amylase and α-Glucosidase Inhibitory Activity of Samples

One of the disorders included in metabolic syndrome are blood glucose disturbances. Large fluctuations of glucose content in the blood are extremely dangerous for patients. In addition to pharmacotherapy of high blood glucose content and insulin resistance, inhibition of glucose release from food is important. In this case, it is important to decrease the activity of enzymes involved in the hydrolysis of polysaccharides. As shown in Table 3, all of the tested samples were characterized by α-amylase and α-glucosidase inhibitor activity. The highest α-amylase inhibitory activity was determined for the ethanolic extract obtained from the jasmonic acid-elicited and freeze-dried plants (EC_50_ = 3.63 mg/mL). It should be noted that this value was not statistically different from samples subjected to the other drying methods.

The highest α-glucosidase inhibitory activity was noted for hydrolysates obtained from freeze-dried control plants (EC_50_ = 1.43 mg/mL). This value showed no significant differences from hydrolysates obtained from the control samples subjected to convection drying (EC_50_ of 1.45 mg/mL). The values are similar to those noted for samples obtained from plants elicited with both elicitors (jasmonic acid and yeast extract) and treated with three drying methods (convection, microwave, and freeze-drying), where the EC_50_ value was in the range from 1.45 to 1.57 mg/mL, and the values did not differ significantly between each other (Table 3).

### 3.4. Antimicrobial Properties

The antimicrobial properties of the samples were tested against bacteria *Escherichia coli* ATCC 25922, *Staphylococcus aureus* ATCC 29737, *Listeria monocytogenes* ATCC BBA-2660, *Bacillus cereus* ATCC 14579, *Salmonella enteritidis* ATCC 4931, and the yeast *Candida albicans* ATCC 90028. The results showed that the samples had certain antimicrobial activity against all tested microorganisms. No antimicrobial properties were detected in the case of samples extracted with PBS. The hydrolysates exhibited no antimicrobial activity against *S. aureus* ATCC 29737 and *S. enteritidis* ATCC 4931. No difference was noted between the control and elicited samples. The MIC values for the bacterial strains tested in the presence of the dried samples were in the range of 1.25–5.0 mg/mL. The highest antimicrobial properties against *C. albicans* ATCC 90028 yeasts was observed for naturally dried ethanolic extracts (MIC = 0.625 mg/mL) as well as ethanolic extracts from freeze-dried and microwave-dried plants (MIC = 1.25–2.5 mg/mL). In the case of bacterial cultures, the highest antibacterial properties against *E. coli* ATCC 25922 and *B. cereus* ATCC 14579 were exhibited by hydrolysates from conventionally dried and freeze-dried yeast extract-elicited plants with MIC and MLC ranging from 1.25 to 2.5 mg/mL (Table 4).

**Figure 1:**
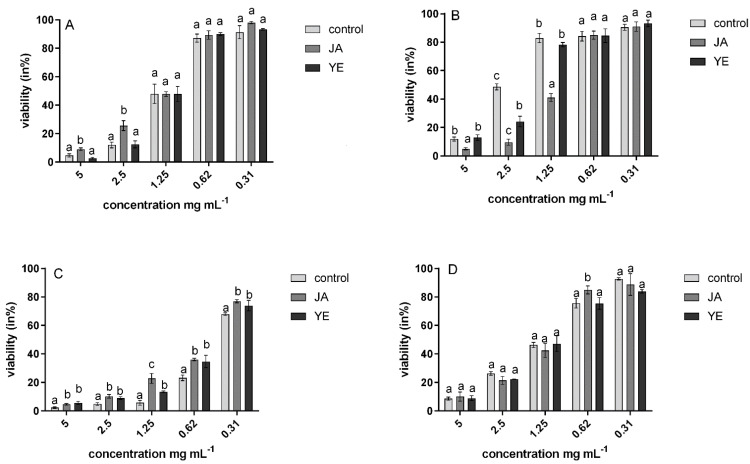
Viability (in %) of yeast *C. albicans* ATCC 90028 against natural (**A**), convention (**B**), microwave (**C**), and freeze-drying samples (**D**).

These results were also confirmed by the resazurin biotoxicity assay. This showed that, in the presence of the samples, the tested microorganisms were characterized by different resazurin reduction rates with the highest value for *C. albicans* ATCC 14579. The highest antimicrobial properties were observed for microwave-dried samples with growth inhibition between 60 and 92% (Figure 1).

## 4. Discussion

Many factors influence the qualitative and quantitative composition of bioactive food compounds. In the case of foods of plant origin, these include growth and cultivation conditions, technological processes, and product storage conditions. Herbs often add a distinctive flavor to foods, giving them fragrance and color. Additionally, they can increase the health-promoting properties of foods. Polyphenols, which exert many health-promoting effects on the organism, are the main group of bioactive compounds in herbs and lovage. Their most important effects are reflected in antioxidant, anti-inflammatory, anti-cancer, or anti-microbial activity. In addition to the factors mentioned above, the content of these compounds is also affected by the type of extraction solvent used, whose choice depends on the chemical structure of the compounds and their interaction with other food compounds [13,14]. In our study, ethanol extraction was more efficient than the PBS-based method. The highest TPC value was determined in the freeze-dried lovage elicited with jasmonic acid (18.48 mg/gDW). This result corresponds well with that reported by Jovanović et al. [15], where ethanolic extracts obtained from wild thyme were characterized by higher TPC than water extracts. In addition, it was shown that the concentration of ethanol used did not affect the content of phenolic compounds, as there was no statistically significant difference between the content of phenolic compounds between 30, 50, and 70% ethanolic extracts. Moreover, the TPC value in ethanolic extracts obtained from *Blepharis linariifolia*, *Cyperus rotundus*, *Guiera senegalensis*, *Maerua pseudopetalosa*, *Tinospora bakis*, and *Dicoma tomentosa* was higher than in water extracts [16].

The highest content of phenolic compounds in our study was found in samples obtained after digestion in conditions simulating the human digestive system (Table 1). Since the enzymes used are mostly proteases, there were protein–phenolic bonds in the samples, and phenolic compounds were released from the food matrix through hydrolysis. The results of our study are consistent with those of the content of phenolic compounds in hydrolysates described by other researchers. The conditions simulating the human digestive system used for hydrolysis also increased the content of phenolic compounds in bean hydrolysates [17], quinoa and djulis sprouts [18], or fruits musts and fruit wines [19].

Additionally, as indicated by the results of our study, the content of phenolic compounds is influenced by the elicitation process and by the method of plant drying. Irrespective of the type of sample, the highest TPC was determined for plants elicited with jasmonic acid. In turn, depending on the type of sample, the content of phenolic compounds was affected by the method of plant drying. Similar results were obtained by Kwaśniewska-Karolak and Mostowski [20], where the effect of the rosemary, sage, and thyme drying method on the content of phenolic compounds was studied. As indicated by the results, TPC was higher for all extracts obtained from lyophilized plants than those subjected to convection drying. Therefore, to obtain dried herbs characterized by a high content of bioactive compounds, attention should also be paid to the method of drying plants.

Phenolic compounds exhibit a variety of bioactive properties, most commonly antioxidant, anti-inflammatory, or anticancer activities. Recently, it has also been noted that these compounds can regulate the activity of the enzymes responsible for the normal functioning of the body. These enzymes influence the pathogenesis of metabolic syndrome. Until recently, this function was mainly attributed to peptides, while it is now known that other groups of food-derived compounds can also affect the activity of these enzymes. A major cause of the development of metabolic syndrome is obesity, which is associated with excessive caloric intake relative to caloric consumption. Additionally, the development of this disease is influenced by the excessive activity of pancreatic lipase, which is responsible for the hydrolysis of 50–70% of fats from food. Therefore, food ingredients that can inhibit excessive pancreatic lipase activity and the development of obesity and support the dietary therapy of the disease are being sought. In our study, not all the samples tested showed inhibitory activity against pancreatic lipase, and the EC_50_ value depended on the elicitor used and the way the plants were dried. The highest lipase inhibitory effect was determined for PBS extract obtained from plants elicited with jasmonic acid and subjected to microwave drying (EC_50_ = 0.12 mg/mL; Table 2). As demonstrated by the results of the study, inhibitory activity is also affected by the type of sample. Ethanol extracts, in many cases, did not show this activity, while PBS extracts had more advantageous properties. Similar results were obtained for 12 different types of fruit, where aqueous extracts had lower IC_50_ values than ethanolic extracts. Among the studied fruits, the best properties were determined for *Chaenomeles japonica* and *Hippophäe rhamnoides* (IC_50_ = 44.88 µg/mL and 59.74 µg/mL, respectively). These values were lower than those determined in our study, which means that the inhibitory activity depends on the type of material.

Obesity is the basis for the development of many other diseases, where hypertension is one of the most common conditions. Excessive ACE activity is also one of the factors enhancing its development. ACE is the main enzyme of the renin-angiotensin-aldosterone system involved in the regulation of blood pressure and responsible for water and electrolyte balance in the organism. ACE converts inactive angiotensin I into angiotensin II, which induces a strong contraction of the muscularis of small blood vessels and significantly raises blood pressure, thereby increasing the heart rate. Angiotensin II also regulates the body’s water and electrolyte homeostasis, stimulation of the sympathetic nervous system, and the biosynthesis and secretion of certain adrenocortical hormones. Excessive ACE activity causes an increase in blood pressure and a decrease in the lumen of blood vessels, which in turn leads to the development of hypertension. Many medications are used to treat hypertension, and despite their efficacy, they cause many side effects. Therefore, compounds, including food ingredients, are being sought to inhibit ACE activity and support the pharmacotherapy of hypertension [21]. To date, much research has focused on peptide ACE inhibitors, but it is now known that other groups of compounds can also inhibit the activity of this enzyme. As in the case of lipase, the PBS extract obtained from the microwave-dried plants elicited with jasmonic acid had the highest inhibitory activity (EC_50_ = 0.16 mg/mL; Table 2). This means that this is the most efficient method for obtaining compounds with potential anti-obesity and antihypertensive activity. Le et al. [22] evaluated the inhibitory activity of 22 different medicinal plants. The study showed that two of them had the highest ACE inhibitory effects: the IC_50_ values of *L. rubra* and *U. sessilifructus* were 1.31 for water extract and 12.86 μg/mL for ethanol extract, respectively. In this case, the type of solvent used to obtain the extracts also influenced their inhibitory activity.

The occurrence of obesity and hypertension is one of the main causes of the development of diabetes, which increasingly afflicts not only adults but also children. The organism needs a constant level of glucose in the blood for proper functioning, while diabetics struggle with abnormal blood glucose levels and wide fluctuations. One therapeutic approach is to inhibit the activity of such enzymes as α-amylase (EC 3.2.1.1) and α-glucosidase (EC 3.2.1.20) responsible for releasing glucose from food and thereby inhibiting the absorption of glucose into the blood. α-amylase causes hydrolysis of α-1,4-glucosidic bonds in starch, resulting in the release of oligosaccharides, which are successively converted into glucose and borderline dextrins. α-Glucosidase, located in the brush border of small intestine enterocytes, causes the hydrolysis of carbohydrates and release of monosaccharides. This is the main contributor to the rise in blood glucose [23]. The administration of an α-glucosidase inhibitor is definitely the most effective strategy in regulating postprandial blood glucose levels and insulin [24]. Therefore, inhibition of the initial hydrolysis of food carbohydrates in the gastrointestinal tract, and the subsequent inhibition of the release of glucose into the blood, play an important role in the prevention of blood glucose disturbances and the development of diabetes. Hence, inhibitors of these enzymes are being sought in food ingredients that can support the pharmacotherapy and dietary management of diabetes. In our study, lower EC_50_ values were determined for α-glucosidase than for α-amylase, and the lowest EC_50_ values were found in samples after hydrolysis in conditions simulating the human digestive system (Table 3). Research indicates that other spices also show potential anti-diabetic properties. These include, e.g., Ceylon cinnamon, cassia cinnamon, sindora pods, or Indian sarsaparilla [25].

Moreover, our study indicated the antimicrobial activity of lovage without and after elicitation, but no significant differences in the antimicrobial properties of lovage without and after elicitation were observed. The highest antimicrobial properties were observed for ethanolic extracts. Recent studies show that lovage extracts have antimicrobial properties [4,26]. Foodborne pathogens are responsible for infections with significant effects on human health, mainly associated with bacterial contamination, especially members of Gram-negative bacteria such as *Salmonella typhi* or *Escherichia coli* and Gram-positive bacteria including *Staphylococcus aureus*, *Listeria monocytogenes*, and *Bacillus cereus* [27]. The *C. albicans* yeasts are commensal microorganisms, colonizing the skin and mucosal surfaces of healthy individuals. However, in some cases, *C. albicans* are associated with opportunistic infection in humans, especially in immunocompromised patients, such as those with HIV/AIDS [28].

The qualitative and quantitative composition of the tested samples influenced their biological activity. In our previous study [29], the content of phenolic acids in individual samples was determined. The results indicated that samples with the highest potential ACE inhibitory properties had the highest content of protocatechuic and caffeic acids. In turn, samples with the highest α -glucosidase inhibitory activity had high concentrations of protocatechuic acid and p-hydroxybenzoic acids.

## 5. Conclusions

Herbs are a rich source of bioactive compounds, mainly phenolic compounds. They have a number of functions, including antioxidant and antimicrobial activity and the inhibition of metabolic syndrome. Their content can be modulated by applying appropriate technological processes, e.g., drying plants or using appropriate elicitors, which contribute to increasing the production of secondary metabolites by plants. Food bioactive compounds undergo many transformations during digestion in the digestive system, which may influence the release of compounds with high biological properties. The present study indicates that the use of lovage can not only enhance the taste and aroma of dishes but also increase their biological potential.

## Figures and Tables

**Table 1:** Total phenolic content (TPC) in the samples.

	Elicitor	C	JA	YE
Drying Method				
Ehanolic extract (mg/gDW)				
natural		11.47 ± 2.16 ^aA^	12.69 ± 1.53 ^aA^	18.09 ± 0.97 ^bB^
convection		16.35 ± 1.30 ^abB^	17.92 ± 1.72 ^bB^	14.12 ± 1.83 ^aA^
microwave		14.56 ± 1.41 ^abAB^	17.58 ± 2.25 ^bB^	13.22 ± 0.82 ^aA^
freeze-drying		14.20 ± 0.75 ^aAB^	18.48 ± 1.66 ^bB^	15.76 ± 1.13 ^aAB^
PBS extract (mg/gDW)				
natural		11.93 ± 1.29 ^aAB^	11.87 ± 0.25 ^aB^	9.99 ± 0.25 ^aA^
convection		9.46 ± 3.02 ^aA^	14.52 ± 1.00 ^bAB^	12.31 ± 1.68 ^abA^
microwave		11.70 ± 0.44 ^aAB^	12.48 ± 0.44 ^aA^	12.49 ± 1.53 ^aA^
freeze-drying		14.18 ± 1.21 ^bB^	13.77 ± 0.30 ^bAB^	10.59 ± 0.6 ^aA^
Hydrolysates (mg/gDW)				
natural		19.98 ± 1.5 ^bA^	21.35 ± 0.89 ^bA^	15.90 ± 2.12 ^aA^
convection		22.34 ± 0.89 ^aA^	22.62 ± 0.78 ^aAB^	21.91 ± 2.65 ^aB^
microwave		21.50 ± 1.74 ^bA^	24.96 ± 0.83 ^cC^	18.44 ± 0.49 ^aAB^
freeze-drying		22.25 ± 0.58 ^bA^	23.76 ± 1.67 ^bBC^	18.77 ± 1.50 ^aAB^

C—control samples without elicitation; JA—samples elicited with 10 µM jasmonic acid; YE—samples elicited with 0.1% yeast extract. All values are the mean ± standard deviation for triplicate experiments. Different capital letters in the same columns indicate significant differences for the same drying method in the same type of sample (ethanolic extract, PBS extract, or hydrolysates) (α = 0.05). Different lowercase letters in the same row indicate significant differences for the same drying method (α = 0.05). The data were analyzed with a one-way analysis of variance (ANOVA), followed by Tukey’s multiple comparison procedure.

**Table 2:** ACE and lipase inhibitory activity of samples.

Sample		C		JA		YE	
	Inhibitor of Enzyme	ACE	Lipase	ACE	Lipase	ACE	Lipase
Drying Method							
Ehanolic Extract (EC_50_ mg/mL)							
natural		0.43 ± 0.06 ^aA^	nd	0.18 ± 0.02 ^aA^	nd	0.61 ± 0.06 ^bA^	nd
convection		1.32 ± 0.05 ^aB^	nd	0.96 ± 0.01 ^aB^	nd	nd	0.15 ± 0.008 ^A^
microwave		0.37 ± 0.01 ^abA^	0.17 ± 0.01 ^a^	0.49 ± 0.03 ^bAC^	0.51 ± 0.06 ^bA^	0.25 ± 0.03 ^aB^	nd
freeze-drying		0.41 ± 0.04 ^aA^	nd	0.61 ± 0.03 ^bC^	0.25 ± 0.03 ^aB^	0.38 ± 0.02 ^aC^	0.24 ± 0.04 ^aB^
PBS Extract (EC_50_ mg/mL)							
natural		nd	0.22 ± 0.03 ^aAB^	nd	0.44 ± 0.06 ^bB^	nd	0.17 ± 0.01 ^aA^
convection		nd	0.14 ± 0.008 ^aA^	nd	0.20 ± 0.01 ^aA^	nd	0.44 ± 0.05 ^bB^
microwave		0.25 ± 0.01 ^b^	0.18 ± 0.01 ^aB^	0.16 ± 0.03 ^a^	0.12 ± 0.02 ^bA^	0.30 ± 0.01 ^b^	0.23 ± 0.02 ^aAB^
freeze-drying		nd	0.26 ± 0.05 ^aAB^	nd	0.19 ± 0.01 ^aA^	nd	0.22 ± 0.01 ^aAB^
Hydrolysates (EC_50_ mg/mL)							
natural		nd	0.17 ± 0.005 ^aA^	nd	0.47 ± 0.07 ^bA^	nd	0.47 ± 0.02 ^bB^
convection		nd	0.69 ± 0.04 ^aB^	1.14 ± 0.03 ^A^	0.67 ± 0.01 ^aA^	nd	0.26 ± 0.006 ^bA^
microwave		3.87 ± 0.85 ^a^	0.69 ± 0.04 ^bB^	0.74 ± 0.02b ^B^	0.75 ± 0.01 ^bA^	0.51 ± 0.04 ^bA^	0.44 ± 0.05 ^aB^
freeze-drying		nd	0.83 ± 0.09 ^cB^	1.32 ± 0.12 ^aA^	0.57 ± 0.06 ^bA^	0.63 ± 0.03 ^bA^	0.30 ± 0.03 ^aA^

C—control samples without elicitation; JA—samples elicited with 10 µM jasmonic acid; YE—samples elicited with 0.1% yeast extract, Nd—not detected. All values are the mean ± standard deviation for triplicate experiments. Different capital letters in the same columns indicate significant differences for the same drying method in the same type of sample (α = 0.05). Different lowercase letters in the same row indicate significant differences for the same indicator (α = 0.05). The data were analyzed with a one-way analysis of variance (ANOVA), followed by Tukey’s multiple comparison procedure.

**Table 3:** α-amylase and α-glucosidase inhibitory activity of sample.

Sample		C		JA		YE	
	Inhibition of Enzyme	α-Amylase	α-Glucosidase	α-Amylase	α-Glucosidase	α-Amylase	α-Glucosidase
Drying Methods							
Ehanolic extract (EC_50_ mg/mL)							
natural		4.73 ± 0.20 ^aA^	2.92 ± 0.03 ^bA^	4.79 ± 0.65 ^aA^	2.83 ± 0.03 ^aA^	5.90 ± 0.97 ^aA^	2.83 ± 0.05 ^aA^
convection		14.00 ± 0.73 ^bB^	2.88 ± 0.08 ^aA^	4.01 ± 0.20 ^aA^	2.78 ± 0.05 ^aA^	3.92 ± 0.20 ^aA^	2.82 ± 0.03 ^aA^
microwave		4.34 ± 0.81 ^aA^	3.31 ± 0.17 ^bB^	3.92 ± 0.81 ^aA^	2.84 ± 0.07 ^aA^	4.03 ± 0.66 ^aA^	2.78 ± 0.02 ^aA^
freeze-drying		22.82 ± 2.32 ^cC^	2.78 ± 0.01 ^aA^	3.63 ± 0.81 ^aA^	2.85 ± 0.05 ^aA^	11.88 ± 2.47 ^bB^	2.85 ± 0.08 ^aA^
PBS extract (EC_50_ mg/mL)							
natural		56.43 ± 4.55 ^cB^	nd	26.16 ± 1.19 ^bB^	59.05 ± 9.17 ^bB^	14.89 ± 0.87 ^aA^	nd
convection		23.84 ± 2.72 ^aA^	nd	67.54 ± 1.73 ^bC^	13.59 ± 0.88 ^aA^	24.77 ± 1.01 ^aB^	25.42 ± 3.39 ^bA^
microwave		101.88 ± 7.75 ^bC^	nd	14.33 ± 0.42 ^aA^	73.14 ± 3.55 ^bC^	17.79 ± 2.3 ^aA^	nd
freeze-drying		18.13 ± 2.01 ^bA^	32.96 ± 3.33 ^b^	11.32 ± 2.32 ^aA^	17.36 ± 0.94 ^aA^	16.68 ± 0.12 ^abA^	26.38 ± 3.68 ^bA^
Hydrolysates (EC_50_ mg/mL)							
natural		17.25 ± 3.22 ^bB^	1.98 ± 0.12 ^aB^	11.80 ± 0.25 ^aA^	1.77 ± 0.24 ^aA^	10.89 ± 0.59 ^aA^	1.94 ± 0.08 ^aB^
convection		10.17 ± 0.83 ^aA^	1.45 ± 0.01 ^aA^	11.35 ± 0.22 ^aA^	1.49 ± 0.04 ^aA^	21.15 ± 2.97 ^bB^	1.57 ± 0.04 ^aA^
microwave		22.95 ± 1.31 ^bC^	1.89 ± 0.01 ^bB^	21.96 ± 1.65 ^bB^	1.54 ± 0.07 ^aA^	12.50 ± 2.12 ^aA^	1.45 ± 0.04 ^aA^
freeze-drying		11.07 ± 1.12 ^aA^	1.43 ± 0.02 ^aA^	10.82 ± 1.57 ^aA^	1.45 ± 0.06 ^aA^	16.46 ± 1.13 ^bB^	1.55 ± 0.02 ^bA^

C—control samples without elicitation; JA—samples elicited with 10 µM jasmonic acid; YE—samples elicited with 0.1% yeast extract; Nd—not detected. All values are the mean ± standard deviation for triplicate experiments. Different capital letters in the same columns indicate significant differences for the same drying method in the same type of sample (α = 0.05). Different lowercase letters in the same row indicate significant differences for the same indicator (α = 0.05). The data were analyzed with a one-way analysis of variance (ANOVA), followed by Tukey’s multiple comparison procedure.

**Table 4:** Antimicrobial activity of dry samples (nd –not detected).

	Microorganism	*E. coli* ATCC 25922	*S. aureus* ATCC 29737	*S. enterica* ATCC 4931	*B. cereus* ATCC 14579	*L. monocytogenes* ATCC BAA-2660	*C. albicans* ATCC 90028
Sample							
Dry Method	Elicitors	MIC/MLC (mg mL^−1^)					
Ethanolic extracts							
natural	control	2.5/5.0	5.0/>5.0	2.5/5.0	2.5/5.0	2.5/5.0	1.25/2.5
	JA	2.5/5.0	5.0/>5.0	2.5/5.0	2.5/5.0	2.5/5.0	1.25/2.5
	YE	2.5/5.0	5.0/>5.0	5.0/>5.0	2.5/5.0	2.5/5.0	1.25/2.5
convection	control	2.5/5.0	2.5/5.0	5.0/>5.0	5.0/>5.0	2.5/5.0	2.5/5.0
	JA	2.5/5.0	2.5/5.0	2.5/5.0	5.0/>5.0	2.5/5.0	1.25/2.5
	YE	2.5/5.0	2.5/5.0	5.0/>5.0	5.0/>5.0	2.5/5.0	2.5/5.0
microwave	control	2.5/5.0	2.5/5.0	2.5/5.0	5.0/>5.0	2.5/5.0	0.62/1.25
	JA	2.5/5.0	2.5/5.0	2.5/5.0	5.0/>5.0	2.5/5.0	0.62/1.25
	YE	2.5/5.0	2.5/5.0	2.5/5.0	5.0/>5.0	2.5/5.0	0.62/1.25
freeze-drying	control	2.5/5.0	2.5/5.0	2.5/5.0	2.5/5.0	2.5/5.0	1.25/2.5
	JA	2.5/5.0	2.5/5.0	2.5/5.0	2.5/5.0	2.5/5.0	1.25/2.5
	YE	2.5/5.0	2.5/5.0	2.5/5.0	2.5/5.0	2.5/5.0	1.252.5
PBS extracts							
natural	control	nd	nd	nd	nd	nd	nd
	JA	nd	nd	nd	nd	nd	nd
	YE	nd	nd	nd	nd	nd	nd
convection	control	nd	nd	nd	nd	nd	nd
	JA	nd	nd	nd	nd	nd	nd
	YE	nd	nd	nd	nd	nd	nd
microwave	control	nd	nd	nd	nd	nd	nd
	JA	nd	nd	nd	nd	nd	nd
	YE	nd	nd	nd	nd	nd	nd
freeze-drying	control	nd	nd	nd	nd	nd	nd
	JA	nd	nd	nd	nd	nd	nd
	YE	nd	nd	nd	nd	nd	nd
Hydrolysates							
natural	control	2.5/5.0	nd	nd	2.5/5.0	5.0	5.0/>5.0
	JA	2.5/5.0	nd	nd	5.0/>5.0	nd	5.0/>5.0
	YE	2.55.0	nd	nd	5.0/>5.0	nd	5.0/>5.0
convection	control	1.25/2.5	nd	nd	2.5/5.0	5.0	1.25/2.5
	JA	5.0/>5.0	nd	nd	5.0/>5.0	nd	5.0/>5.0
	YE	1.25/2.5	nd	nd	2.5/5.0	nd	1.25/2.5
microwave	control	5.0/>5.0	nd	nd	1.25/2.5	5.0	5.0/>5.0
	JA	5.0/>5.0	nd	nd	2.5/5.0	nd	1.25/2.5
	YE	5.0/>5.0	nd	nd	2.5/5.0	nd	1.25/2.5
freeze-drying	control	1.25/2.5	nd	nd	2.5/5.0	nd	5.0/>5.0
	JA	5.0/>5.0	nd	nd	5.0/>5.0	nd	5.0/>5.0
	YE	1.25/2.5	nd	nd	1.25/2.5	5.0	5.0/>5.0

## Data Availability

Not applicable.
